# A 3,5-dinitropyridin-2yl substituted naphthalimide-based fluorescent probe for the selective detection of biothiols and its application in cell-imaging†

**DOI:** 10.1039/d1ra00010a

**Published:** 2021-03-01

**Authors:** Yihua Zhuo, Yanyu Zhang, Yadong Feng, Yuqing Xu, Qihua You, Lei Zhang, Huabin Huang, Lili Lin

**Affiliations:** College of Environment and Public Health, Xiamen Huaxia University 288 Tianma Road, Jimei District Xiamen 361024 P. R. of China jyyqh@hxxy.edu.cn; Biochemical Pharmacy Engineering Research Center of Fujian Province University 288 Tianma Road Jimei District Xiamen 361024 P. R. of China; School of Physics and Optoelectronics Engineering, Ludong University Yantai 264025 P. R. of China; Biology Institute of Shanxi 50 Shifan Road, Xiaodian District Taiyuan 030006 P. R. of China

## Abstract

A naphthalimide-based fluorescent probe was developed for the sensitive and selective detection of biothiols. The fluorescence of the probe was quenched by the electron-withdrawing 3,5-dinitropyridin-2-yl group *via* the photoinduced electron transfer process, and turned on by biothiol-triggered nucleophilic aromatic substitution. The sensing mechanism was confirmed by HPLC analysis and theoretical calculations. The probe shows a satisfactory response time of 30 min with low detection limits (Cys: 0.32 μM; Hcy: 0.88 μM; GSH: 0.46 μM). Furthermore, the probe was successfully utilized to detect endogenous and exogenous biothiols in HeLa cells.

## Abstract 2

A highly selective OFF–ON fluorescent probe has been developed for the sensing of biothiols with a satisfactory response time and low detection limit. Also, the probe can be successfully applied for the sensing of biothiols in living cells.

## Graphical abstract

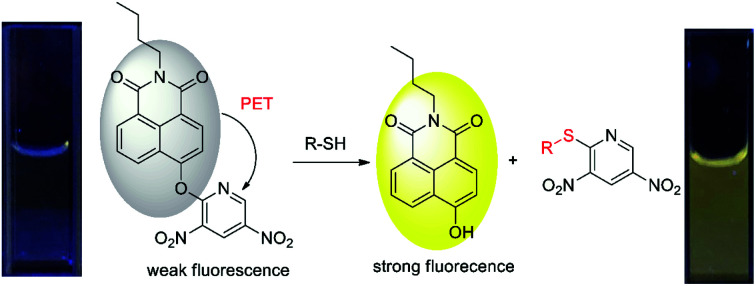

## 1. Introduction

Biological thiols (biothiols), including cysteine (Cys), homocysteine (Hcy), and glutathione (GSH), are found to be closely associated with many diseases.^1,2^ For example, an abnormal level of Cys is relevant to skin lesions, liver damage, brain injury, and Parkinson's disease.^3^ A high concentration of Hcy has been reported to be linked to cardiovascular disease, osteoporosis, and Alzheimer's disease.^4–6^ As the most abundant biothiol and an important antioxidant in cells, the decrease of GSH is associated with neurodegeneration, inflammation, and so forth.^7–9^ Therefore, the development of highly selective and sensitive detection methods for biothiols is important for the early diagnosis of diseases.

Fluorescent probes have attracted wide attention due to their high selectivity and sensitivity, real-time detection, non-invasiveness, and biocompatibility characteristics.^10–13^ In the development of a fluorescent probe for biothiol sensing, the design strategies are mainly focused on Michael addition,^14–16^ nucleophilic cleavage-cyclization,^17–19^ metal coordination complex-displacement,^20–22^ and nucleophilic aromatic substitution (SNAr).^23–26^ Giving the strong nucleophilicity of biothiols, especially their corresponding deprotonated thiolate anion, it is desirable to introduce a strong electron-withdrawing group as a biothiol recognition site to a fluorophore platform. The introducing group also provides a quenching effect *via* photoinduced electron transfer (PET) mechanism. Hence, a probe can be designed with high reactivity and sensitivity toward biothiols. Recently, 3,5-dinitropyridin-2-yl was chosen as a biothiol recognition site due to its higher electron-withdrawing ability than other commonly used groups such as 2,4-dinitrophenyl, 2,4-dinitrobenzenesulfonyl and 7-nitro-2,1,3-benzoxadiazole group.^24^ This biothiol recognition group exhibited satisfactory selectivity and sensitivity toward biothiols in aqueous buffer and living cells. Therefore, it is necessary to extend the application of this recognition group to improve its sensing ability in the design of a biothiol-targeting fluorescent probe.

In this work, we report a new fluorescent probe for the detection of biothiols based on the nucleophilic aromatic substitution. *N*-Butyl-4-hydroxy-1,8-naphthalimide (NAP-OH) was selected as the fluorophore due to its high photostability, and good biocompatibility.^27,28^NAP-DNP exhibits high selectivity and sensitivity for biothiols with low detection limits (0.32 μM, 0.88 μM and 0.46 μM for Cys, Hcy and GSH, respectively) and medium response (30 min). The proposed recognition mechanism was corroborated by HPLC analysis and theoretical calculations. The low cell cytotoxicity indicates that NAP-DNP is suitable for the sensing of biothiols in living cells.

## 2. Results and discussion

### 2.1. The synthesis of NAP-DNP

**Scheme 1:**
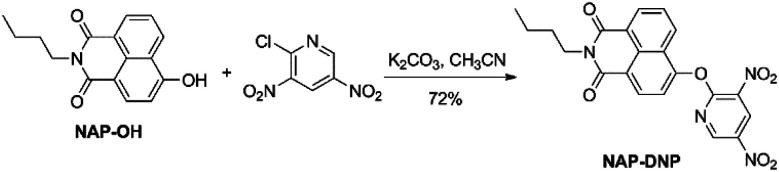
Synthetic route to probe NAP-DNP.

Probe NAP-DNP was synthesized by the reaction of NAP-OH with 2-chloro-3,5-dinitropyridine in a 72% yield, as shown in Scheme 1. The product was fully characterized by ^1^H NMR, ^13^C NMR, and HRMS analysis. Detailed synthetic procedure and structure characterizations are given in the experimental section and the ESI (Fig. S1–S3†).

### 2.2. The selectivity of NAP-DNP for biothiols

**Fig. 1:**
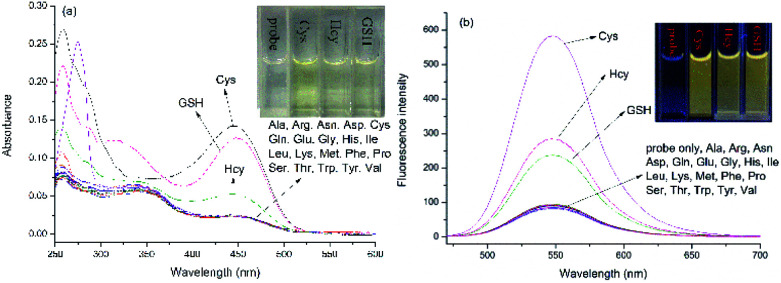
UV-Vis absorption (a) and fluorescence (b) spectra of NAP-DNP (10 μM) in DMSO–Tris buffer (1 : 9, v/v, 20 mM, pH = 7.4) upon addition of different amino acids (200 μM). Inset: color of solution (a) and fluorescence (b) changes of NAP-DNP without and with different biothiols (Cys, Hcy and GSH).

**Fig. 2:**
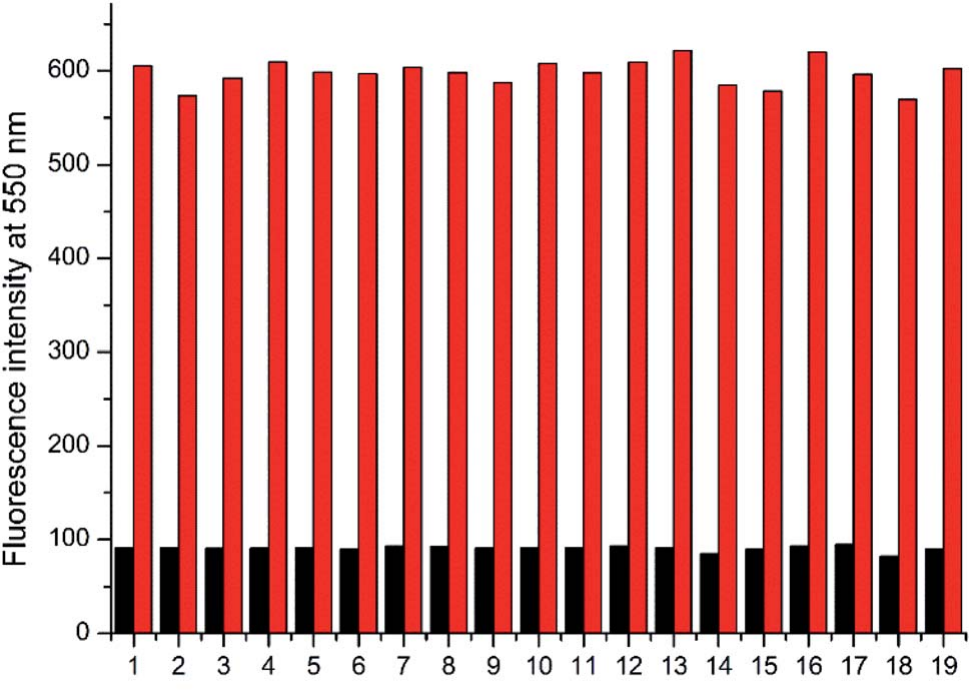
Fluorescence intensity changes of NAP-DNP (10 μM) for Cys in the presence of various amino acids (200 μM) in DMSO–Tris buffer (1 : 9, v/v, 20 mM, pH = 7.4) for 30 min (*λ*_ex_/*λ*_em_ = 450/550 nm). Black bars represent the addition of a single analyte including (1) Ala; (2) Arg; (3) Asn; (4) Asp; (5) Gln; (6) Glu; (7) Gly; (8) His; (9) Ile; (10) Leu; (11) Lys; (12) Met; (13) Phe; (14) Pro; (15) Ser; (16) Thr; (17) Trp; (18) Tyr; (19) Val. Red bars represent the subsequent addition of Cys (200 μM) to the mixture.

The absorption and emission responses of NAP-DNP (10 μM) toward various amino acids were investigated in Tris buffer (20 mM, pH 7.4, containing 10% DMSO, v/v). As shown in Fig. 1, NAP-DNP exhibited a weak absorption band centered at 450 nm in the visible range and a weak emission band centered at 550 nm. Upon addition of biothiols (Cys, Hcy and GSH), the absorbance and fluorescence intensity increase to some extent (7.0-, 2.8- and 3.4-fold enhancement in fluorescence intensity for Cys, Hcy and GSH, respectively). Meanwhile, the solution of NAP-DNP changed from colorless to pale yellow (inset of Fig. 1a) and emitted distinct lemon-yellow fluorescence under UV lamp (inset of Fig. 1b). In contrast, other amino acids including Ala, Asn, Asp, Arg, Gln, Glu, Gly, His, Ile, Leu, Lys, Met, Phe, Pro, Ser, Thr, Trp, Tyr and Val, induced negligible change in absorbance and fluorescence intensity of NAP-DNP. Also, the presence of the abovementioned amino acids (200 μM) has no significant effect on the sensing ability of NAP-DNP to biothiol (Fig. 2). These results indicate that NAP-DNP is a highly selective fluorescent probe for biothiols.

### 2.3. The sensitivity of NAP-DNP for biothiols

**Fig. 3:**
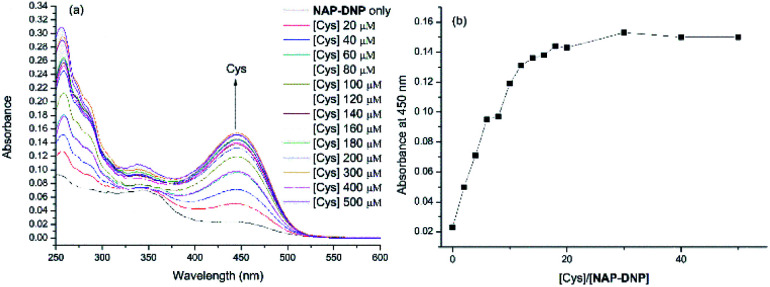
(a) UV-Vis absorption titration of NAP-DNP (10 μM) in DMSO–Tris buffer (1 : 9, v/v, 20 mM, pH = 7.4) upon addition of Cys. (b) Plot of absorbance at 450 nm of NAP-DNP (10 μM) as a function of Cys concentration.

**Fig. 4:**
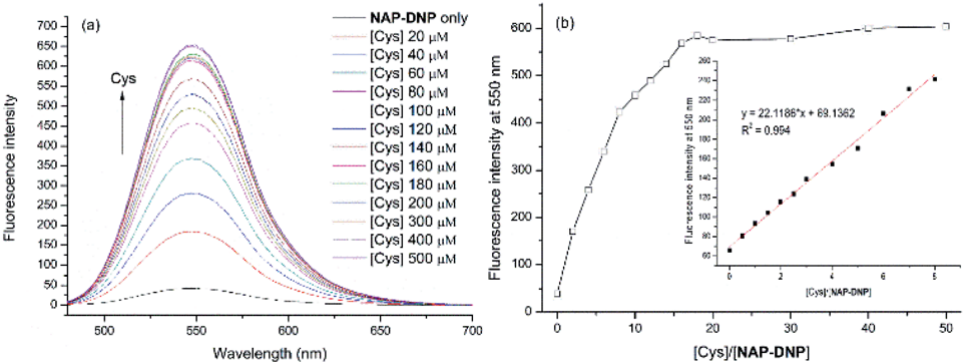
(a) Fluorescence titration of NAP-DNP (10 μM) in DMSO–Tris buffer (1 : 9, v/v, 20 mM, pH = 7.4) upon addition of Cys. Excitation wavelength is set at 460 nm. Excitation/emission wavelength slit = 5/3 nm. (b) Plot of fluorescence intensity at 550 nm of NAP-DNP (10 μM) as a function of Cys concentration. Inset: plot of the linear relationship (*R*^2^ = 0.994) between the fluorescence intensity at 550 nm of NAP-DNP (5 μM) and Cys concentration (0, 0.5, 1.0, 1.5, 2.0, 2.5, 3.0, 4.0, 5.0, 6.0, 7.0, 8.0 equiv.).

To investigate the sensing ability of NAP-DNP for biothiols (Cys as the representative), titration experiments were conducted with UV-Vis and fluorescence spectroscopy. As shown in Fig. 3a and 4a, upon addition of Cys, the absorption peak at 450 nm and fluorescence intensity at 550 nm increased gradually and reached a plateau after the addition of 200 μM of Cys (Fig. 3b and 4b). A linear relationship (*R*^2^ = 0.994) was found between the fluorescence intensity at 550 nm and Cys concentration in the range of 0–40 μM (inset of Fig. 4b), suggesting that NAP-DNP is capable of sensing biothiols both qualitatively and quantitatively. The detection limit (3*σ*/*k*) of NAP-DNP for Cys, Hcy and GSH was measured to be 0.32 μM, 0.88 μM and 0.46 μM, respectively (inset of Fig. 4b, S4 and S5†). The values are lower than that of many reported biothiol probes, indicating that NAP-DNP is highly sensitive to biothiols.

### 2.4. The effect of pH and time-dependent response

**Fig. 5:**
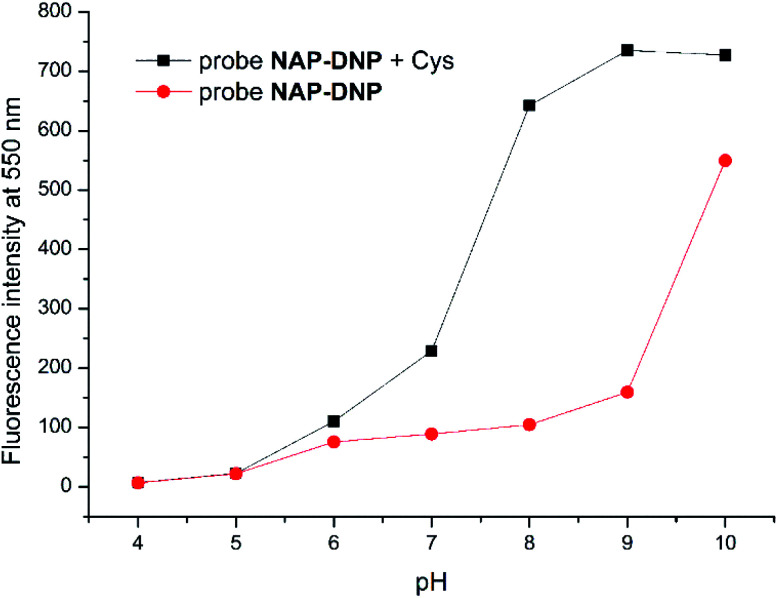
Fluorescence intensity at 550 nm of probe NAP-DNP (10 μM) in DMSO–Tris buffer (1 : 9, v/v, 20 mM, pH = 7.4) without (red circle) and with (black square) addition of Cys (200 μM) as a function of pH.

**Fig. 6:**
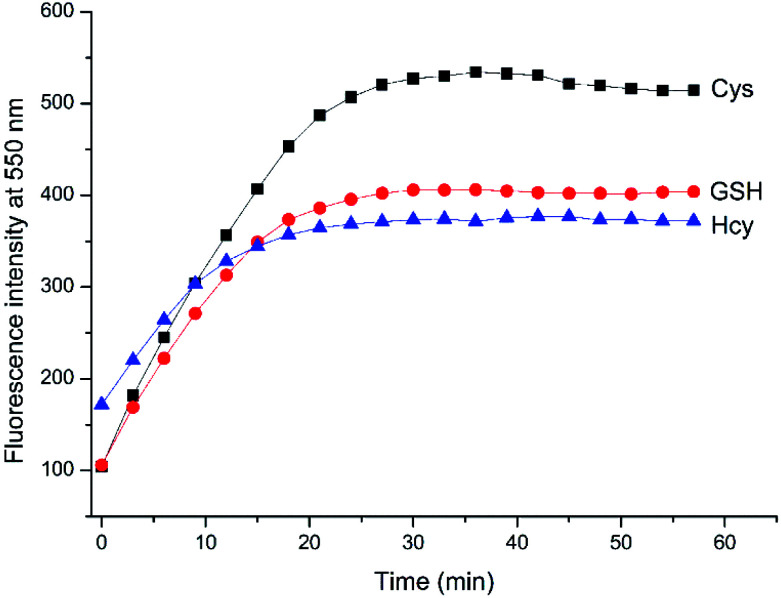
Time-dependent fluorescence enhancement at 550 nm of NAP-DNP (10 μM) in DMSO–Tris buffer (1 : 9, v/v, 20 mM, pH = 7.4) upon the addition of various Cys, Hcy and GSH (200 μM), respectively.

To evaluate the sensing pH range for NAP-DNP, fluorescent detection of Cys at different pH values was investigated. As shown in Fig. 5, NAP-DNP exhibits a very weak fluorescence under strong or moderate acidic conditions (pH < 5), and a slight enhancement in fluorescence intensity over a pH range from 6 to 8. However, a significant fluorescence enhancement was observed at pH over 9. These results may be ascribed to the strong electron-withdrawing 3,5-dinitropyridin-2-yl group facilitating the nucleophilic attack by OH^−^. In the presence of Cys (200 μM), the fluorescence intensity of NAP-DNP increased gradually when the pH increased from 5 to 9 and reached a steady reading at pH over 9. Considering the ratio of fluorescence intensity of NAP-DNP with Cys to the fluorescence intensity of NAP-DNP at different pH values (Fig. S6†) and further application in cell-imaging, physiological pH at 7.4 was selected throughout the experiments. The time-dependent responses of NAP-DNP to biothiols were also investigated (Fig. 6). It was found that the fluorescence output of NAP-DNP became steady after 30 min addition of biothiols at pH 7.4. Therefore, all of the experiments were measured after the addition of biothiols for 30 min.

### 2.5. Sensing mechanism

**Fig. 7:**
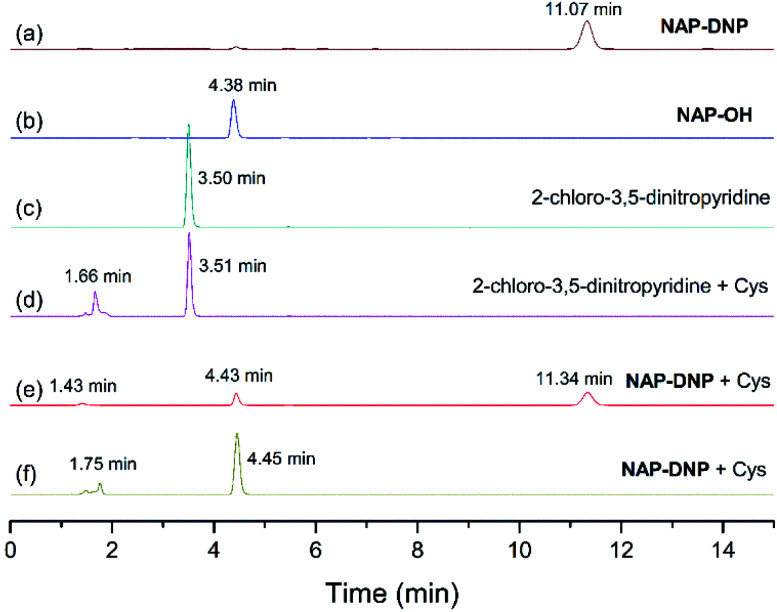
The reversed-phase HPLC with absorption (254 nm) detection. (a) Probe NAP-DNP. (b) Compound NAP-OH. (c) 2-Chloro-3,5-dinitropyridine. (d) The reaction mixture of 2-chloro-3,5-dinitropyridine and Cys. (e) The reaction mixture of NAP-DNP and Cys for 15 minutes. (f) The reaction mixture of NAP-DNP and Cys for 30 minutes.

**Scheme 2:**
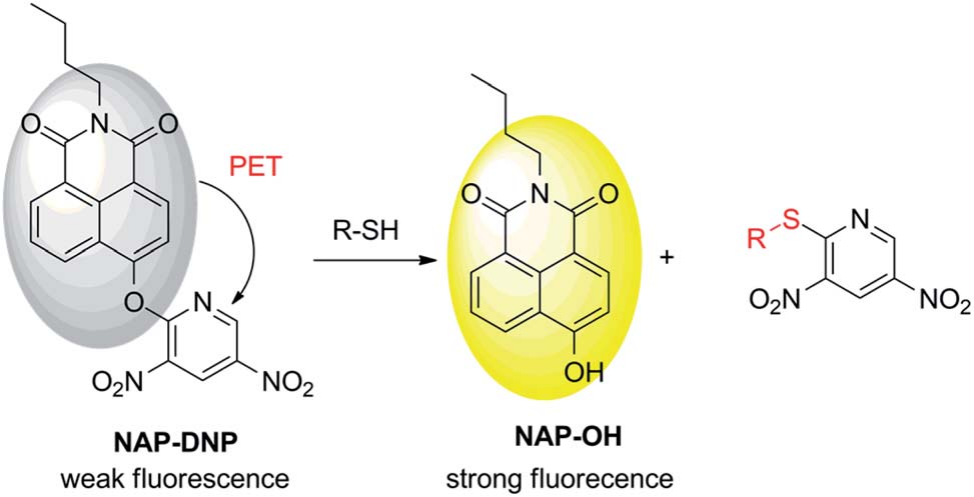
Proposed mechanism for sensing of biothiols.

To test the hypothesis on the biothiol-triggered activation of NAP-DNP, the reaction of NAP-DNP with Cys was analyzed *via* HPLC. As shown in Fig. 7, NAP-DNP, NAP-OH and 2-chloro-3,5-dinitropyridine displayed a single peak with a retention time at 11.07, 4.38 and 3.50 min, respectively. Upon the addition of Cys to the solution of NAP-DNP for 15 min, the peak at 11.34 min weakened while peaks at 1.43 and 4.43 min emerged (Fig. 7e). After reaction for 30 min, the peak at 11.34 min disappeared while peaks at 1.75 and 4.45 min strengthened (Fig. 7f). The peak at 1.75 min is in accordance with the result of the reaction of 2-chloro-3,5-dinitropyridine with Cys (Fig. 7d). This result confirmed the proposed sensing mechanism (Scheme 2) that the fluorescence enhancement of NAP-DNP in the presence of Cys is ascribed to the release of molecule NAP-OH from NAP-DNP.

### 2.6. Theoretical calculations

**Fig. 8:**
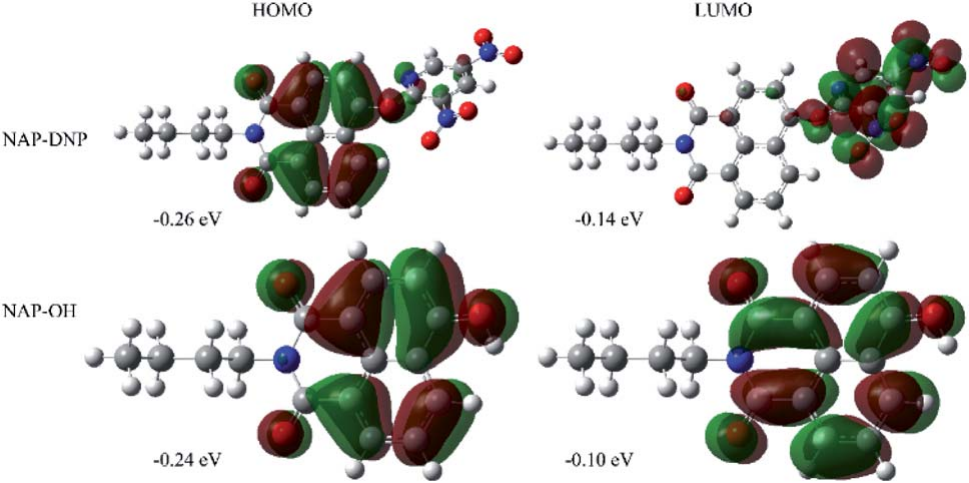
Electron density distributions in the HOMO and LUMO states of NAP-DNP and NAP-OH calculated by DFT in Gaussian 16, C.01 program.

To prove our speculation of the proposed sensing mechanism, density functional theory (DFT)-based theoretical calculations were carried out using Gaussian 16, C.01 program.^29^ The energy-optimized geometries and electronic structure of NAP-DNP and NAP-OH were generated by using B3LYP/6-31G(d) basis set, respectively. The computational results revealed that in NAP-DNP, the highest occupied molecular orbital (HOMO) was mainly located on the electron-donating naphthalimide moiety, whereas the lowest unoccupied molecular orbital (LUMO) was located primarily on the 3,5-dinitropyridin-2-yl moiety (Fig. 8). The energy level of LUMO and HOMO demonstrated that electrons could transfer from the naphthalimide moiety to the 3,5-dinitropyridin-2-yl moiety when NAP-DNP was excited, which corresponds to the fluorescence quenching of NAP-DNP. However, when NAP-DNP was converted to NAP-OH by biothiol, both the HOMO and LUMO were mainly located on the naphthalimide moiety. Therefore, the electron transfer process was inhibited and the fluorescence was in the “turn-on” state. These results are in reasonable agreement with the experimental results.

### 2.7. Imaging of biothiols in living cells

Encouraged by the above experimental results, a further application of NAP-DNP for the sensing of biothiols in living cells was conducted. Initially, we evaluated the cytotoxicity of NAP-DNP at various concentrations using the CCK-8 assay (Fig. S7†). Living HeLa cells were incubated with different concentrations of NAP-DNP (0, 5, 10, 20, 50 μM) for 24 h at 37 °C, with the results suggesting that NAP-DNP has very low cytotoxicity to HeLa cells, even at high concentration.

**Fig. 9:**
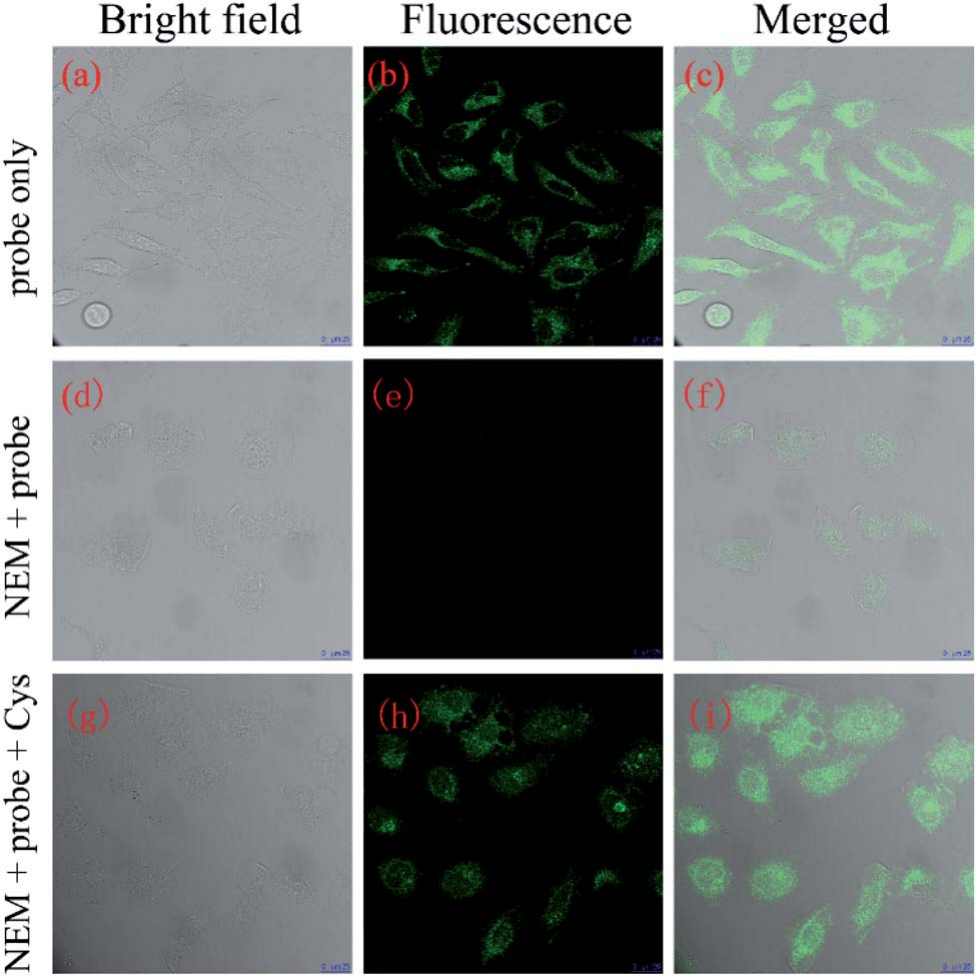
Confocal microscopy images of HeLa cells. (a–c) HeLa cells incubated with probe NAP-DNP (10 μM) for 30 min; (d–f) HeLa cells pretreated with NEM (5 mM) for 30 min and further incubated with NAP-DNP (10 μM); (g–i) HeLa cells pretreated with NEM (5 mM) for 30 min and then incubated with NAP-DNP (10 μM) and Cys (10 mM) for 30 min (a, d and g): bright field image; (b, e and h): fluorescence image collected in the range of 500–600 nm; (c, f and i): overlap of bright field and fluorescence image. Excitation wavelength: 488 nm scale bar: 25 μm.

The reaction time of NAP-DNP for biothiols was determined to be 30 min, which indicated that NAP-DNP is suitable for the real-time detection of biothiols in living cells. When HeLa cells were incubated with NAP-DNP (10 μM) at 37 °C for 30 min, strong fluorescence was observed (Fig. 9a–c). Considering that the high concentrations of intracellular biothiols (Cys: 30–200 μM; Hcy: 5–12 μM; GSH: 1–20 mM),^30,31^ it could be concluded that the fluorescence enhancement was mainly caused by the reaction of NAP-DNP with intracellular biothiols. As a control, no fluorescence was observed for cells pre-incubated with *N*-ethylmaleimide (NEM, a thiol scavenger) (Fig. 9d–f). Furthermore, when HeLa cells were successively treated with NEM, NAP-DNP and Cys, a bright fluorescence was observed (Fig. 9g–i). These experiments demonstrated that NAP-DNP can be utilized as an efficient tool to monitor exogenous and endogenous biothiols in living cells.

## 3. Conclusions

In summary, we have successfully developed a turn-on fluorescent probe NAP-DNP for the sensing of biothiols based on the nucleophilic aromatic substitution mechanism. The probe displays high selectivity to discriminate between biothiols and other amino acids. Moreover, the probe shows a satisfactory response time of 30 min with low detection limits (Cys: 0.32 μM; Hcy: 0.88 μM; GSH: 0.46 μM). In particular, the sensing mechanism of the probe to biothiols was investigated *via* HPLC analysis and theoretical calculations. Furthermore, the probe shows very low cytotoxicity to living cells and has been successfully used for the detection of endogenous and exogenous biothiols.

## 4. Experimental

### 4.1. Synthesis of probe NAP-DNP

To a solution of compound NAP-OH (270 mg, 1.0 mmol) and 2-chloro-3,5-dinitropyridine (205 mg, 1.0 mmol) in anhydrous CH_3_CN (10 mL) was added K_2_CO_3_ (280 mg, 2.0 mmol) in one portion. Then the reaction mixture was stirred overnight at room temperature under N_2_ atmosphere. The reaction mixture was concentrated under reduced pressure, and the residue was purified by silica gel column chromatography (PE/EtOAc = 5 : 1) as a yellow solid (315 mg, yield 72%). Mp: 169.0–169.5 °C. ^1^H NMR (CDCl_3_, 400 MHz) *δ* = 9.26 (d, *J* = 2.5 Hz, 1H), 9.10 (d, *J* = 2.5 Hz, 1H), 8.69–8.66 (m, 2H), 8.27 (d, *J* = 7.7 Hz, 1H), 7.79 (dd, *J* = 7.4 Hz, *J* = 7.5 Hz, 1H), 7.62 (d, *J* = 8.1 Hz, 1H), 4.20 (t, *J* = 7.5 Hz, 2H), 1.76–1.69 (m, 2H), 1.51–1.41 (m, 2H), 0.99 (t, *J* = 7.4 Hz, 3H) ppm. ^13^C NMR (CDCl_3_, 100 MHz) *δ* = 163.8, 163.2, 157.8, 152.2, 147.5, 140.0, 132.1, 131.7, 131.5, 129.6, 128.0, 127.4, 125.1, 123.2, 121.5, 119.4, 40.4, 30.2, 20.4, 13.8 ppm. HRMS (ESI): *m*/*z* [M + H^+^] calcd for C_21_H_17_N_4_O_7_^+^: 437.1092; found: 437.1086.

## Author contributions

Y. Zhuo and Y. Zhang contributed equally to this work.

## Conflicts of interest

There are no conflicts to declare.

## Supplementary Material

RA-011-D1RA00010A-s001
