# Supplementary material for: A 3,5-dinitropyridin-2yl substituted naphthalimide-based fluorescent probe for the selective detection of biothiols and its application in cell-imaging
Source: RSC Adv. 2021 Mar 1;11(16):9290–5. doi: 10.1039/d1ra00010a (PMC8695333; doi:10.1039/d1ra00010a)
Supplement: RA-011-D1RA00010A-s001 [file RA-011-D1RA00010A-s001.pdf]

Electronic Supplementary Material (ESI) for RSC Advances.
This journal is © The Royal Society of Chemistry 2021

Supporting Information

A 3,5-dinitropyridin-2yl substituted naphthalimide-based fluorescent probe for the selective detection of biothiols and its application in cell-imaging

Yihua Zhuo,[a] Yanyu Zhang,[a] Yadong Feng,[ab] Yuqing Xu,*[c] Qihua You, *[ab] and Lei Zhang[d]

[a] College of Environment and Public Health, Xiamen Huaxia University, 288 Tianma Road, Jimei District, Xiamen 361024, P. R. of China

[b] Biochemical Pharmacy Engineering Research Center of Fujian Province University, 288 Tianma Road, Jimei District, Xiamen 361024, P. R. of China

[c] School of Physics and Optoelectronics Engineering, Ludong University, Yantai 264025, P. R. of China.

[d] Biology Institute of Shanxi, 50 Shifan Road, Xiaodian District, Taiyuan 030006, P. R. of China

E-mail: jyyqh@hxxy.edu.cn

Figure. S1. ^{1}H NMR spectrum of probe **NAP-DNP**.
Figure. S2. ^{13}C NMR spectrum of probe **NAP-DNP**.
Figure S3. HRMS spectrum of probe **NAP-DNP**.
Figure S4. (a) fluorescence titration of **NAP-DNP** (10 μM) in DMSO-Tris buffer (1:9, v/v, 20 mM, pH = 7.4) upon addition of Hcy. Excitation wavelength is set at 460 nm. Excitation/emission wavelength slit = 5/3 nm. (b) Fluorescence intensity at 550 nm of **NAP-DNP** (10 μM) as a function of Hcy.
Figure S5. (a) fluorescence titration of **NAP-DNP** (10 μM) in DMSO-Tris buffer (1:9, v/v, 20 mM, pH = 7.4) upon addition of GSH. Excitation wavelength is set at 460 nm. Excitation/emission wavelength slit = 5/3 nm. (b) Fluorescence intensity at 550 nm of **NAP-DNP** (10 μM) as a function of GSH.
Figure S6. Plot of ratio of fluorescence intensity of **NAP-DNP** with Cys to the fluorescence intensity of **NAP-DNP** at different pH values.
Figure S7. CCK-8 cell viabilities of HeLa cells incubated with **NAP-DNP** for 24 h at different incubation concentration (0.1% DMSO, 0, 5, 10, 20, 50 μM).

Experimental section

1. Materials and instruments

^1H NMR and ^{13}C NMR spectra were recorded on a Bruker Avance-III 400 MHz Spectrometer (at 400 and 100 MHz, respectively). High-resolution mass spectroscopy (HRMS) was performed with an ESI source and a TOF detector. Fluorescence emission spectra and UV-Vis spectra were collected on a SHIMAZU UV-2450 and an RF-5301 spectrometer, respectively. The melting point was determined with a X-5A melting point apparatus (uncorrected). The pH measurements were recorded on a Rex PHS-3G pH/mV/temperature benchtop meter equipped with an E-201-C pH combined electrode. Double-distilled water was used throughout the experiments. Fluorescence imaging was performed by confocal fluorescence microscopy on an Olympus FluoView Fv1000 laser scanning microscope.

All solvents and reagents were obtained from Shanghai Titan Scientific Co. Ltd. and were of analytical grade. Analytical thin-layer chromatography was performed using TLC silica gel 60 GF254 (aluminum sheets, Merck KGaA). L-Alanine (Ala), L-aspartic acid (Asp), L-glutamate (Glu), glycine (Gly), L-lysine (Lys), L-methionine (Met), L-phenylalanine (Phe), L-proline (Pro), L-threonine (Thr), L-(-)-tryptophan (Trp), and L-tyrosine (Tyr) were stored in a vacuum desiccator. L-Cysteine (Cys), DL-homocysteine (Hcy), glutathione (GSH), L-(+)-arginine (Arg), L-asparagine monohydrate (Asn), L-glutamine (Gln), L-histidine (His), L-isoleucine (Ile), L-leucine (Leu), L-serine (Ser), L-valine (Val) and tris(hydroxymethyl)aminomethane (Tris) were stored in refrigerator.

2. Spectroscopic measurements

The absorption and fluorescence experiments were conducted with DMSO-Tris buffer (1:9, v/v, 20 mM, pH = 7.4). Stock solution of probe **NAP-DNP** (1.0 mM) was prepared in DMSO, and the stock solution was diluted to a final concentration of 10 μM in DMSO-Tris buffer (1:9, v/v, 20 mM, pH = 7.4) in a quartz cell for the absorption and fluorescence spectral experiments. Stock solutions (10 mM) of amino acids including Ala, Asn, Asp, Arg, Gln, Glu, Gly, His, Ile, Leu, Lys, Met, Phe, Pro, Ser, Thr, Trp, Tyr, Val, Cys, Hcy and GSH were prepared in Tris buffer (20 mM, pH = 7.4).

In the fluorescence spectral experiments, the excitation wavelength was set at 460 nm, and the excitation and emission slit widths were set at 5 and 3 nm, respectively.

3. HPLC measurement

A Shimazu LC-20A HPLC system equipped with a C18 column (Inertsil ODS-SP, 5mm,150 mm × 4.6 mm) was used. Eluent: 60% CH_3CN (0-15 min). The flow rate was 1.0 mL/min, and the eluents were detected at 254 nm. Injection volume: 10 μL.

4. Cytotoxicity assay

The cellular toxicity of probe **NAP-DNP** was performed using a Cell Counting Kit-8 (CCK-8). HeLa cells were seeded into 96-well plates at a density of 4000/well, cultured at 37 °C with 5% CO_2 for 24 hours, and then different concentrations of probe **NAP-DNP** (5, 10, 20, 50 μM) were added to the wells. Subsequently, 10 μL of CCK-8 was added to each well followed by incubation for an additional 4 hours at 37 °C under 5% CO_2. The absorbance of each well was measured on a micro-plate reader (Tecan, Austria) at a detection wavelength of 450 nm.

The following formula was used to calculate the inhibition of cell growth: Cell viability (%) = (mean of absorbance value of treatment group/mean of absorbance value of control)×100%.

5. Cell imaging

HeLa cells were cultured in DMEM with 10% fetal bovine serum (FBS) at 37°C, 5% CO_2 and 95% humidity. All the cells were washed 3 times with phosphate-buffered saline (PBS) and then incubated with PBS free fresh media for subsequent cell imaging. For the imaging of endogenous biothiols detection, the cells were incubated with probe **NAP-DNP** (10 μM) for 30 min. In the control experiment, HeLa cells were pretreated with *N*-ethylmaleimide (NEM, 5 mM) for 40 min at 37°C followed by washing 3 times with PBS and further incubation with **NAP-DNP** (10 μM) for 30 min. For the imaging of endogenous biothiols detection, the cells were applied with the same treatment as control experiment followed by the incubation with Cys (10 mM) for 30 min. All the cells were washed 3 times with PBS before visualization under a CLSM.

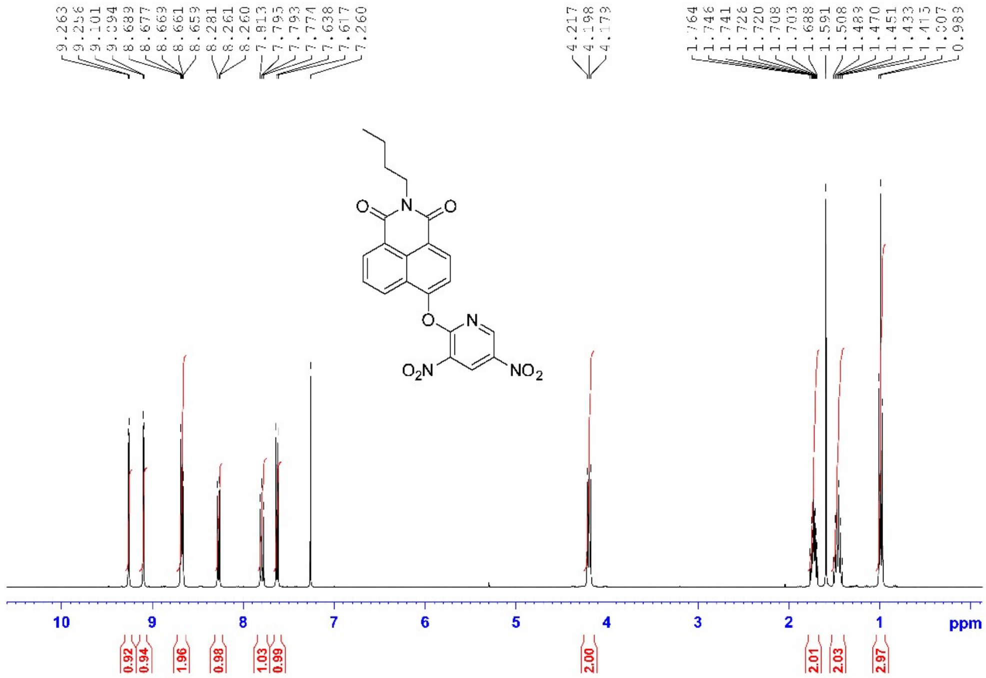

Figure. S1. ^{1}H NMR spectrum of probe **NAP-DNP**.

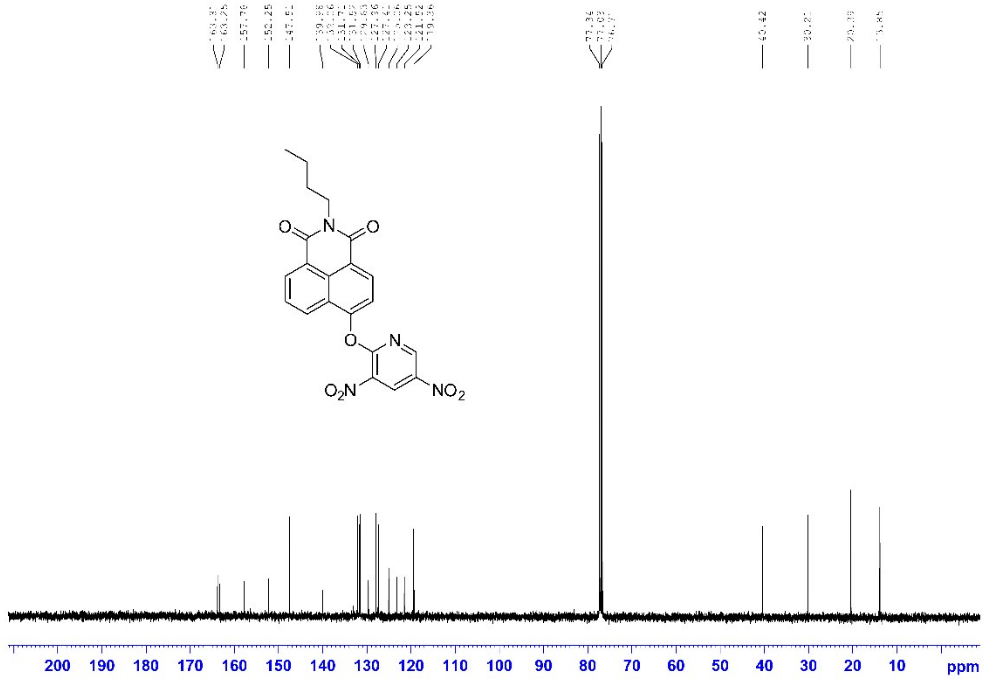

Figure. S2. ^{13}C NMR spectrum of probe **NAP-DNP**.

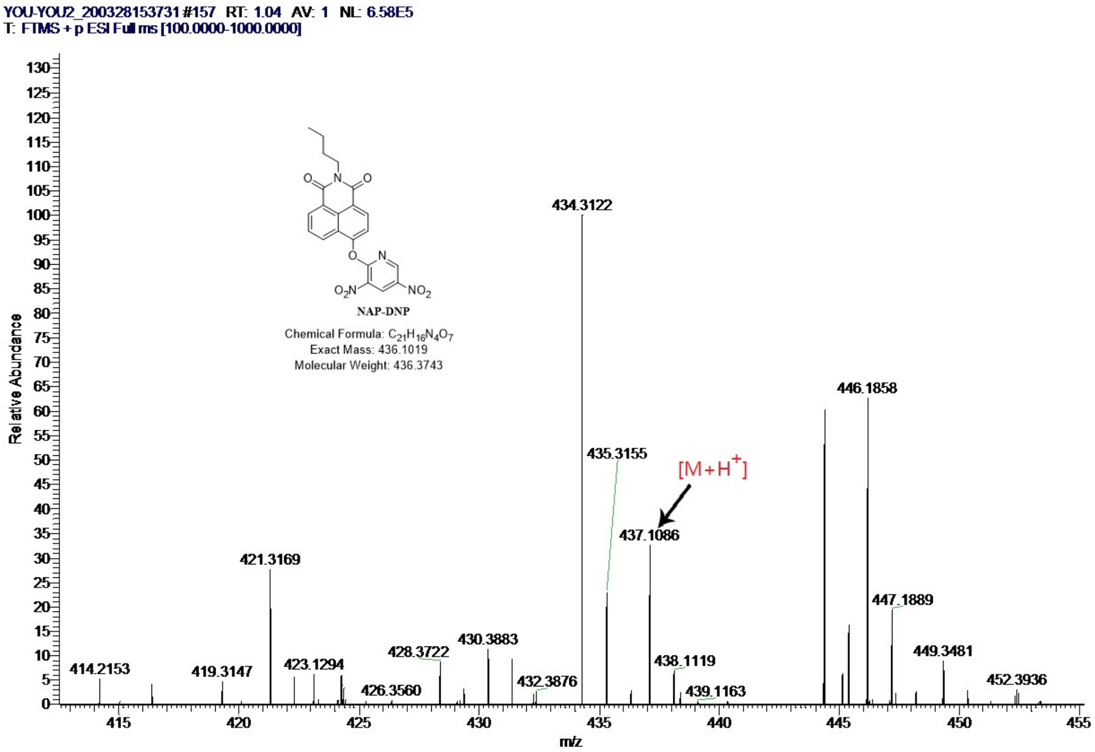

Figure S3. HRMS spectrum of probe **NAP-DNP**.

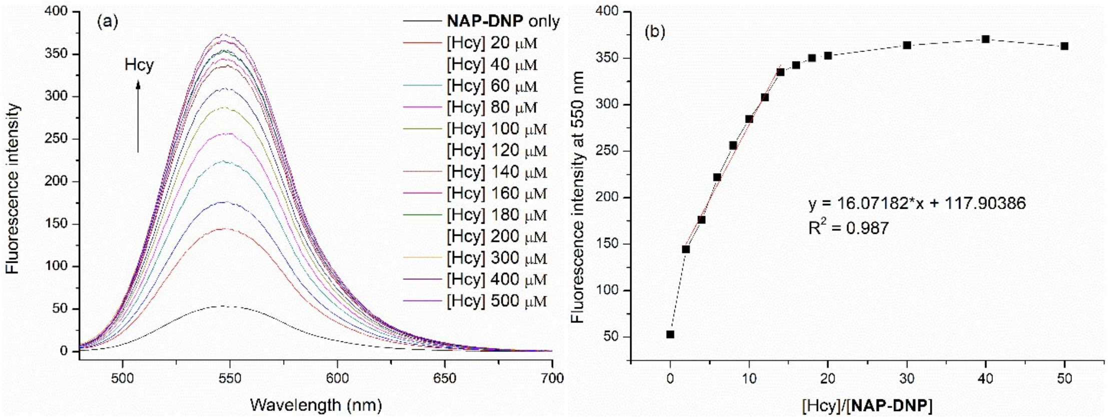

Figure S4. (a) fluorescence titration of **NAP-DNP** (10 μM) in DMSO-Tris buffer (1:9, v/v, 20 mM, pH = 7.4) upon addition of Hcy. Excitation wavelength is set at 460 nm. Excitation/emission wavelength slit = 5/3 nm. (b) Fluorescence intensity at 550 nm of **NAP-DNP** (10 μM) as a function of Hcy.

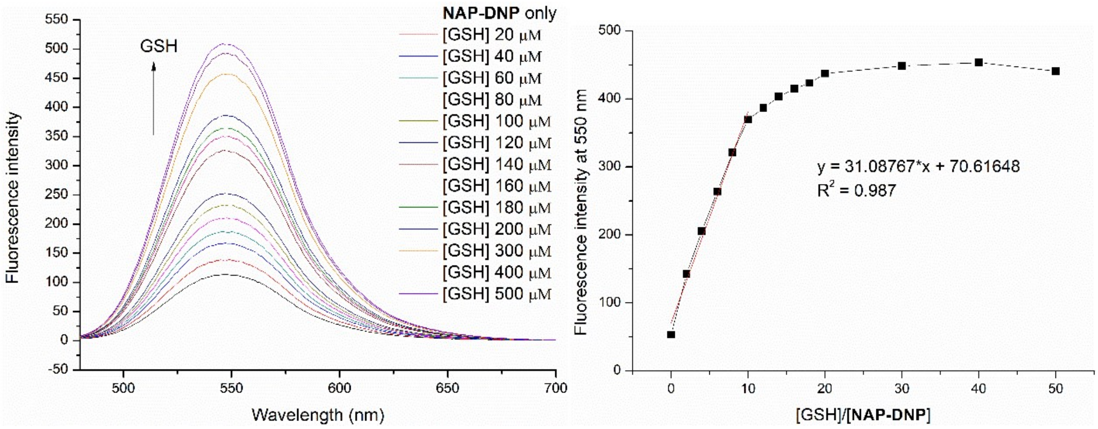

Figure S5. (a) fluorescence titration of **NAP-DNP** (10 μM) in DMSO-Tris buffer (1:9, v/v, 20 mM, pH = 7.4) upon addition of GSH. Excitation wavelength is set at 460 nm. Excitation/emission wavelength slit = 5/3 nm. (b) Fluorescence intensity at 550 nm of **NAP-DNP** (10 μM) as a function of GSH.

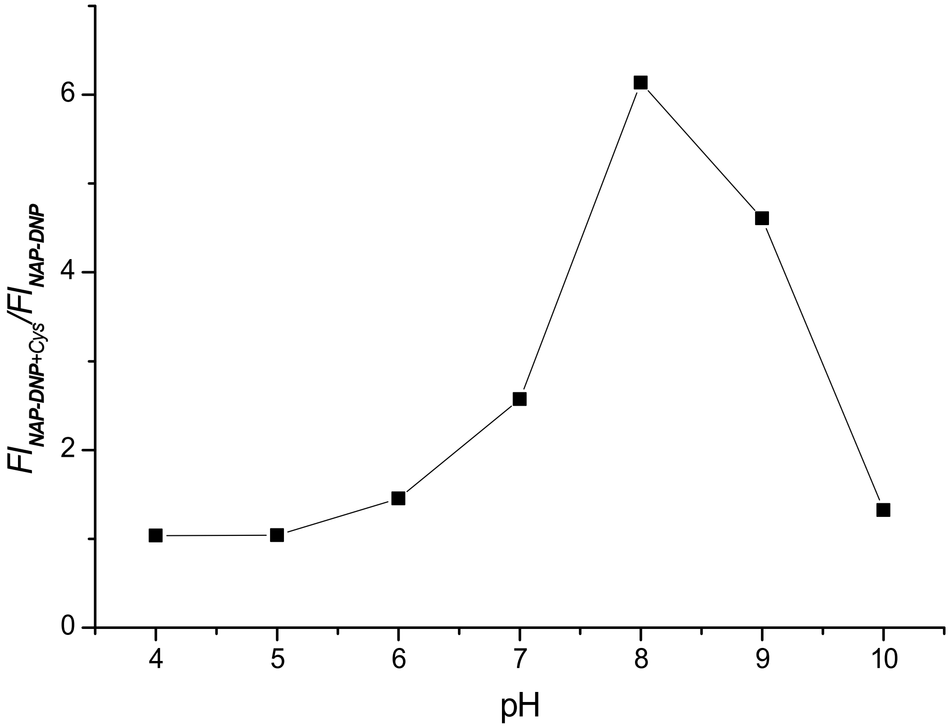

Figure S6. Plot of ratio of fluorescence intensity of **NAP-DNP** with Cys to the fluorescence intensity of **NAP-DNP** at different pH values.

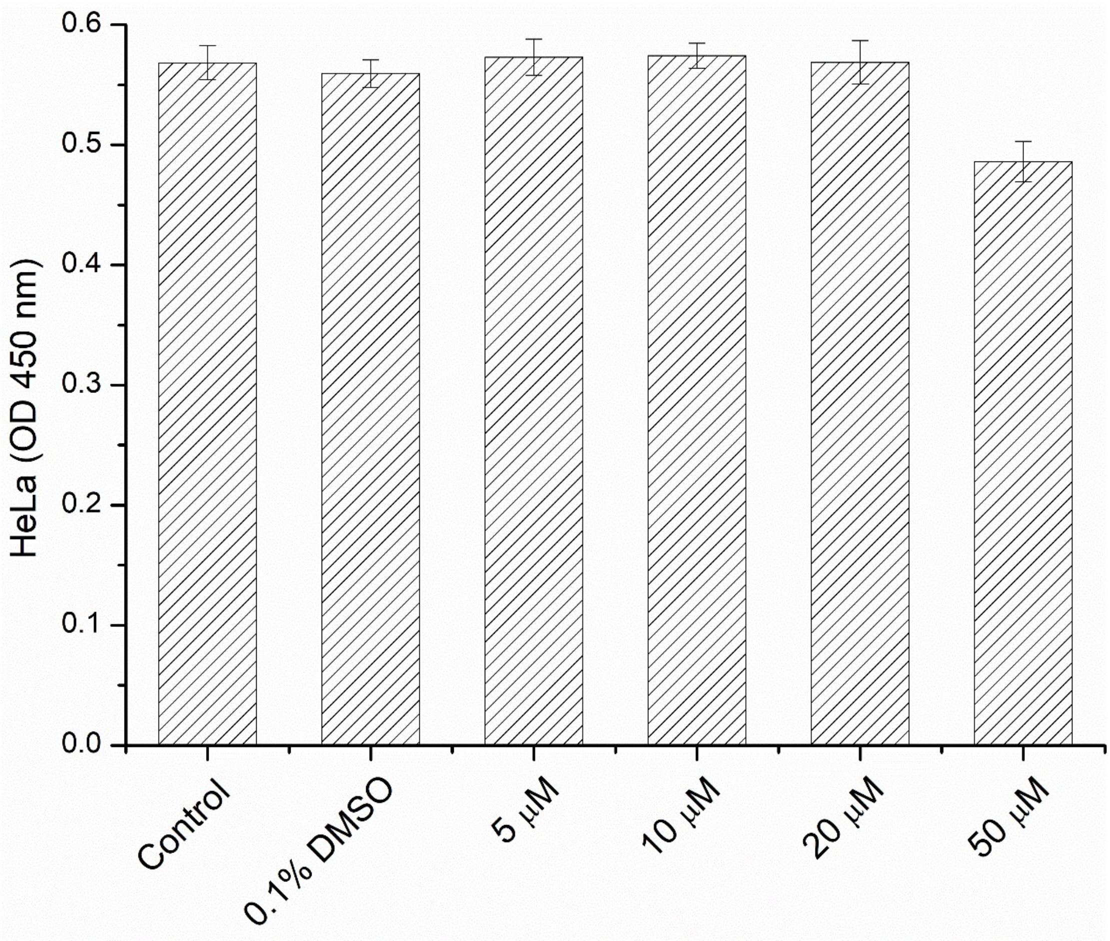

Figure S7. CCK-8 cell viabilities of HeLa cells incubated with **NAP-DNP** for 24 h at different incubation concentration (0.1% DMSO, 0, 5, 10, 20, 50 μM).
